# Endovascular Repair for Abdominal Aneurysm with Concomitant Aortoiliac Vein Fistula Diagnosed by Four-Dimensional Computed Tomography

**DOI:** 10.3400/avd.cr.22-00019

**Published:** 2022-12-25

**Authors:** Chiharu Tanaka, Hidekazu Furuya, Shunsuke Kamei, Satoshi Suda, Masaomi Yamaguchi

**Affiliations:** 1 Department of Cardiovascular Surgery, Tokai University Hachioji Hospital, Hachioji, Tokyo, Japan; 2 Department of Radiology, Tokai University Hachioji Hospital, Hachioji, Tokyo, Japan

**Keywords:** aorto-venous fistula, four-dimensional computed tomography (CT), endovascular repair

## Abstract

A 78-year-old man complaining of left leg swelling was diagnosed with an abdominal aortic aneurysm with an irregular margin. A four-dimensional computed tomography (CT) showed an aortoiliac vein fistula. An AFX stent graft was urgently implanted, and a Viabahn VBX was inserted into the left iliac vein. The aneurysmal sac was embolized. After the procedure, enhanced CT confirmed a patent stent graft without any endoleak or fistula. The patient was discharged ambulatory. An aortoiliac vein fistula is a differential diagnosis for leg edema, and a four-dimensional CT is beneficial in diagnosing the condition.

## Introduction

An abdominal aortic aneurysm (AAA) with an aorto-venous fistula (AVF) is uncommon. Aorto-caval fistulas are found in 0.22–6.04% of infrarenal AAA cases,^1)^ and aortoiliac vein fistulas are even less common. In AVF cases, 68% have an aorto-caval fistula, and 20% have an aorto-left iliac vein fistula.^2)^ Herein, we report a case of AAA with an aorto-left iliac vein fistula diagnosed using four-dimensional computed tomography (4DCT).

## Case Report

**Figure 1:**
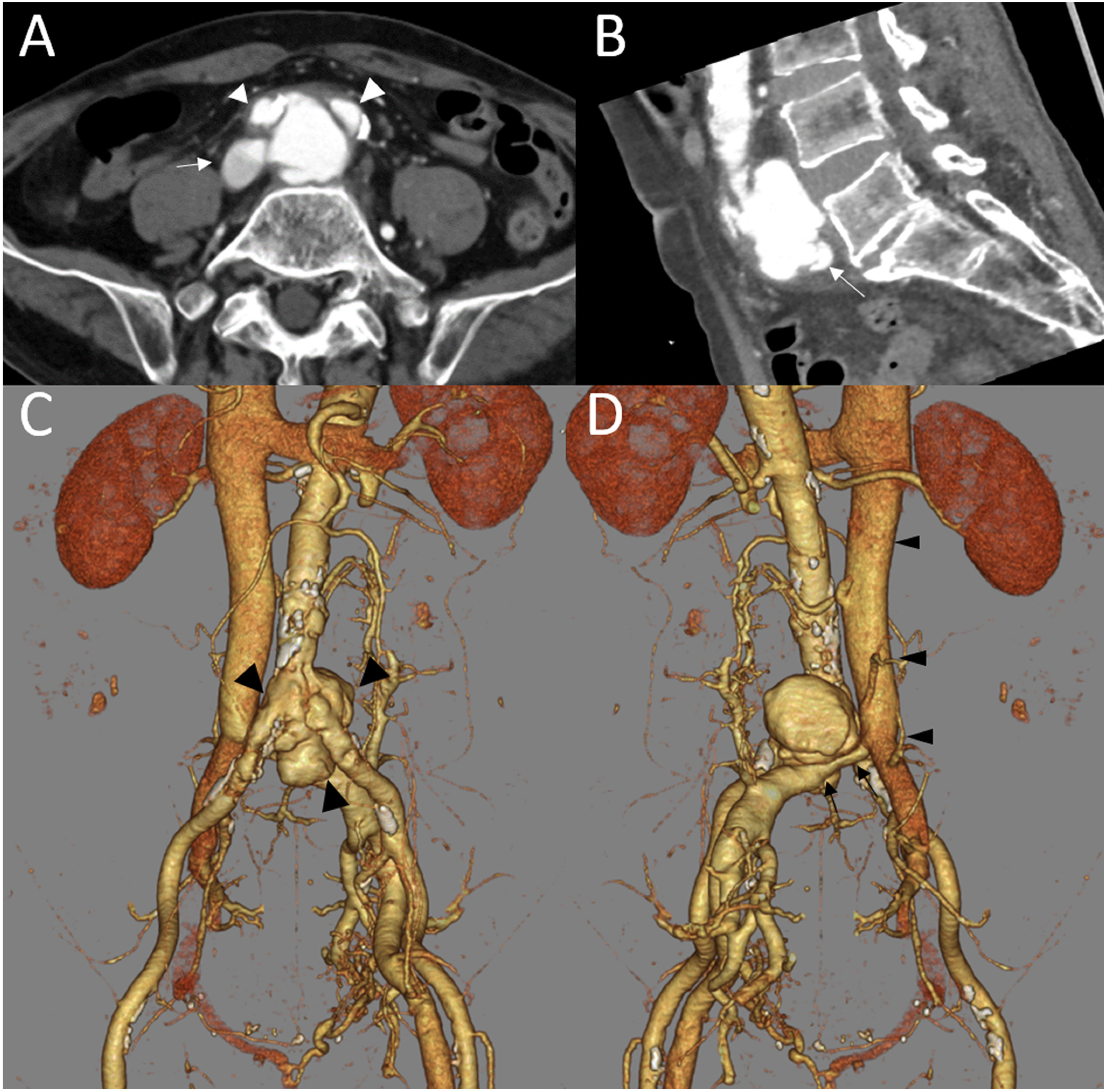
Fig. 1 Preoperative computed tomography (CT) findings. (**A**) Axial view of the abdominal aortic aneurysm (AAA). The AAA has a diameter of 41 mm and an irregular margin. The arrowheads indicate bilateral common iliac arteries, and the arrow indicates the left common iliac vein, which was enhanced in the early phase. (**B**) Sagittal view of the AAA extracted from the four-dimensional CT findings. The arrow points to the left iliac vein, which is compressed by the aneurysm and has a fistula communicating with the AAA. (**C**) Three-dimensional CT findings from the ventral aspect. The aneurysm is located at the level of the aortic bifurcation, as indicated by the arrowheads. (**D**) Three-dimensional CT findings from the dorsal aspect. The left iliac vein and inferior vena cava are enhanced at the same time as the aorta, as indicated by the arrowheads. The left common iliac vein is compressed by the aneurysm, as indicated by the arrows.

A 78-year-old man complained of left leg swelling without pain for 3 days. A history of hypertension, cerebral infarction, dementia, and gastric ulcers was noted. He also suffered from spinal canal stenosis, resulting in gait difficulty. Blood tests revealed a high white blood cell count of 11.7×10^9^/µL, high C-reactive protein of 34.3 nmol/L, and mild anemia (hemoglobin of 106 g/L). Enhanced computed tomography (CT) (320 slice, Aquilion PRISM Edition, Canon Medical Systems Co., Otawara, Japan) showed an AAA 41 mm in diameter with an irregular margin (**Figs. 1A** and **1B**). The left common iliac vein and inferior vena cava were enhanced in the early phase (**Figs. 1C** and **1D**). The 4DCT demonstrated the AAA and its fistula communicating with the left common iliac vein without massive bleeding in the retroperitoneum (Supplemental data). Echocardiography showed normal left ventricular function, mildly high tricuspid regurgitant pressure gradient of 41 mmHg, and a normal inferior vena cava diameter of 15 mm with respiratory fluctuation.

**Figure 2:**
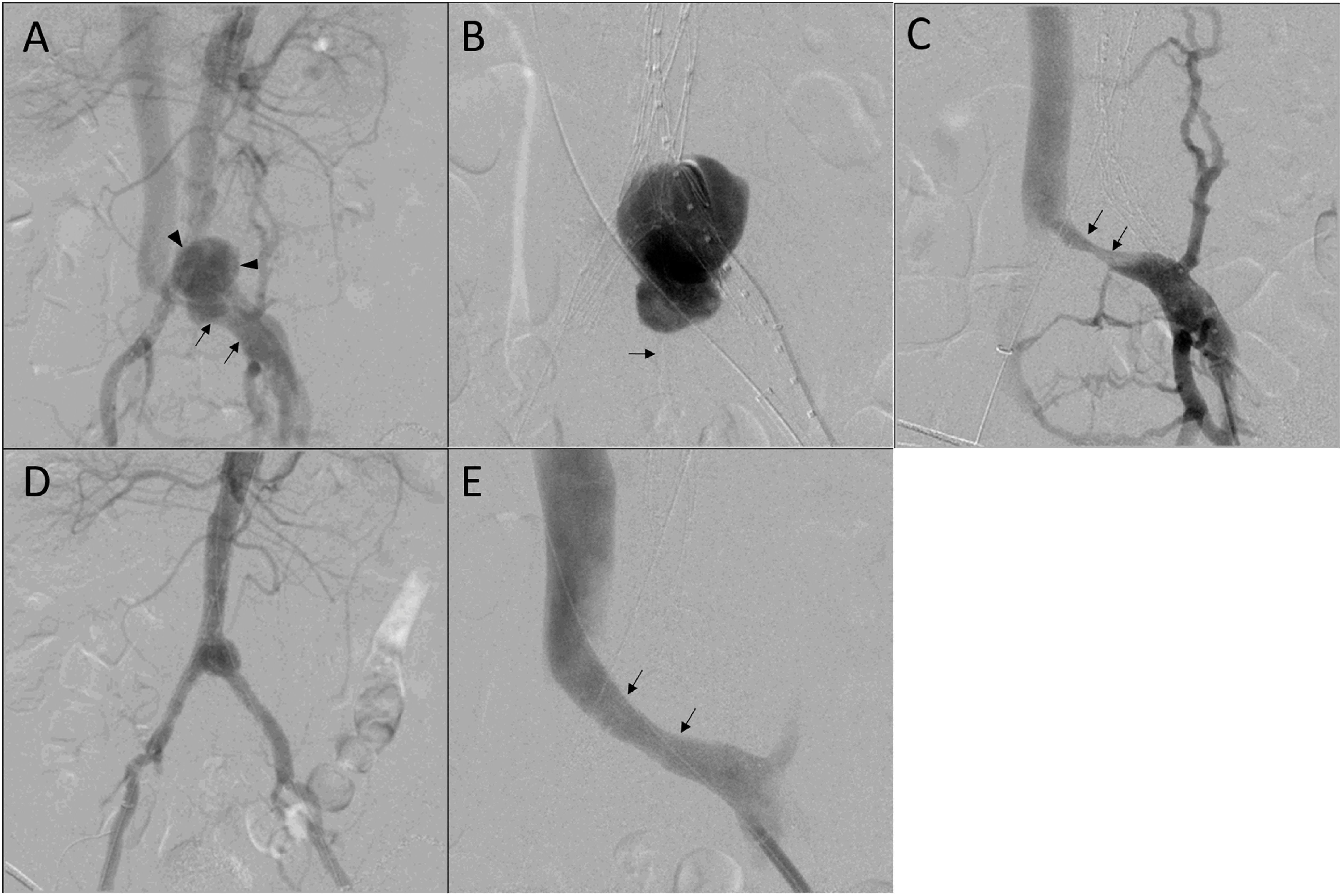
Fig. 2 Findings of the angiography. (**A**) Aortography before stent graft insertion. The abdominal aortic aneurysm (AAA) and the left common iliac vein are enhanced, as indicated by the arrowheads and arrows, respectively. The inferior vena cava is also enhanced. (**B**) Aneurysmal sac imaging. The median sacral artery is enhanced indistinctly, as indicated by the arrow, and was not identified intraoperatively. (**C**) Venography before stent graft insertion. The left common iliac vein is compressed by the aneurysm, as indicated by the arrows. The fistula communicating with the aneurysm is not recognized. (**D**) Aortography after stent graft insertion. The AAA is covered without any obvious endoleak, and the left iliac vein and inferior vena cava are not enhanced. (**E**) Venography after stent graft insertion. There is expansion of the left common iliac vein, as indicated by the arrows.

An AFX BEA22-70/I16-40 stent graft (Endologix LLC, Irvine, CA, USA) was urgently implanted. Before insertion, aortography revealed the AAA and enhancement of the left iliac vein and inferior vena cava (**Fig. 2A**). To prevent endoleak, Impede-FX (Shape Memory Medical Inc., Santa Clara, CA, USA) was used to plug the aneurysmal sac during insertion of the AFX stent graft. The sac imaging described the AAA and the median sacral artery, which appeared indistinctly and was not identified intraoperatively (**Fig. 2B**). Venography revealed that the left iliac vein was compressed by the aneurysm without recognition of the fistula (**Fig. 2C**). A Viabahn VBX (BXA105901J, W. L. Gore & Associates, Inc., Newark, DE, USA) was inserted into the left iliac vein to seal the perforated wall and expand the inner lumen. After insertion of the stent grafts, aortography demonstrated the AAA covered by the AFX stent graft without visible endoleak (**Fig. 2D**), and venography showed an expansion of the left iliac vein (**Fig. 2E**). The procedure was performed without any complications.

**Figure 3:**
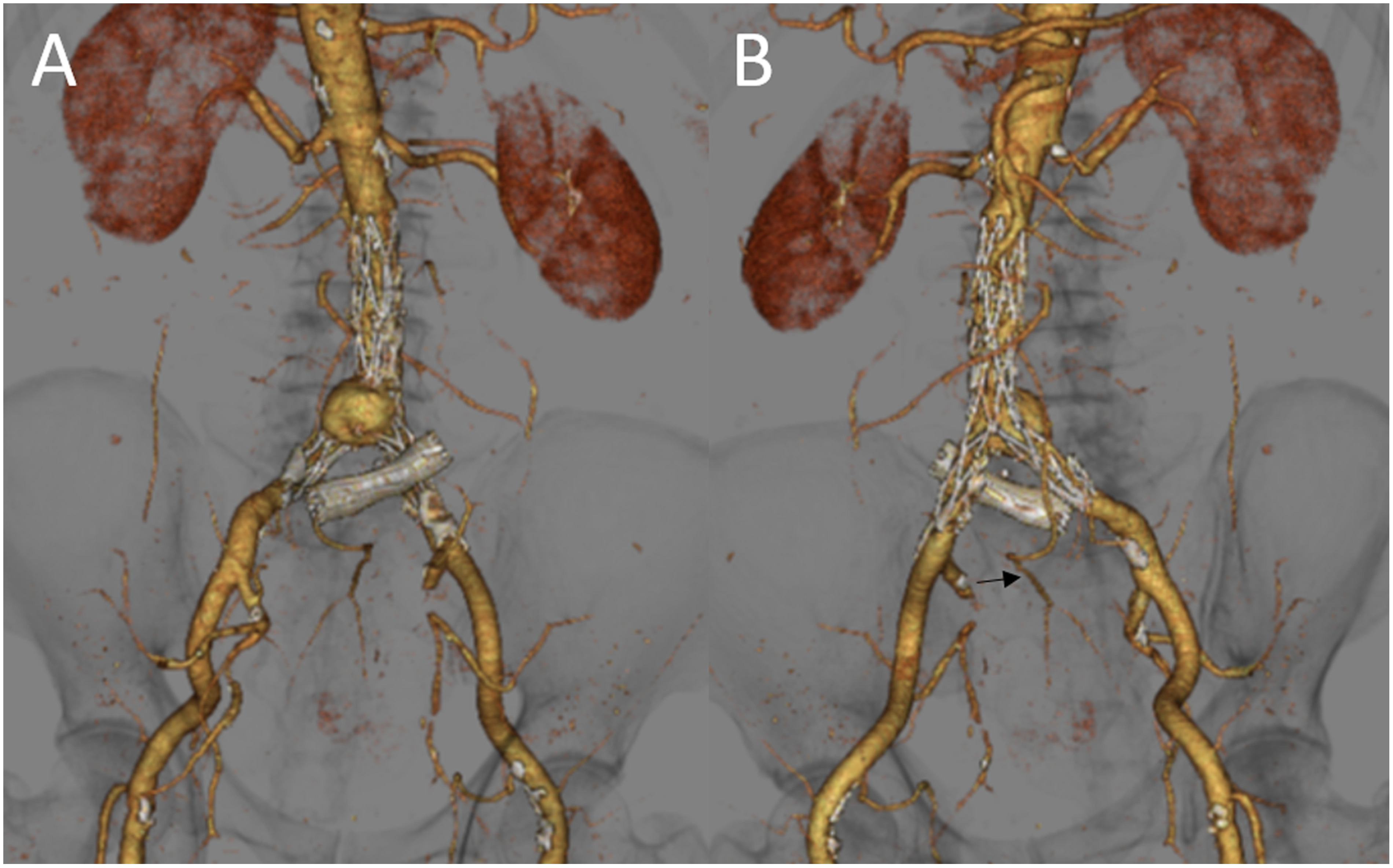
Fig. 3 Postoperative three-dimensional computed tomography (CT) findings. (**A**) Findings from the ventral aspect. The aneurysm and left iliac vein are covered with stent grafts without any fistula. (**B**) Findings from the dorsal aspect. The median sacral artery is fed by the inferior mesenteric artery, as indicated by the arrow.

One week after the surgery, enhanced CT confirmed that the aneurysm was covered by the stent graft without any endoleak and revealed the patent left iliac vein, which was not enhanced in the early phase (**Figs. 3A** and **3B**). A short segment of the left anterior tibial artery was occluded due to embolus scattered from the aneurysm. Because the patient had no symptoms, cilostazol and limaprost were added without any invasive treatment. On postoperative day 13, the patient was discharged ambulatory.

## Discussion

We encountered an AAA with AVF, which was diagnosed using 4DCT and repaired via endovascular technique in an emergency setting. Maeda et al. reported that all AAA with AVF cases presented with abdominal or back pain, 60% of cases were in shock, and 80% of cases had heart failure.^3)^ Our case only presented with left leg edema due to an isolated aortoiliac vein fistula without bleeding into the retroperitoneal space. This is an uncommon etiology of leg edema, and an isolated AVF is estimated to occur in only 0.4% of AAA cases.^1)^ Therefore, although uncommon, AAA with AVF can be a differential diagnosis of leg edema. Additionally, the average diameter of an AAA with AVF was reported to be 7.1–13.0 cm.^3)^ The diameter of the present case was 4.1 cm, but it had irregular margins, suggesting two possibilities: (1) its irregular margin can trigger AVF formation, and (2) the aneurysm originated from the median sacral artery, not from the aorta. A median sacral artery aneurysm is an extremely rare condition without any trauma or iatrogenic reason,^4)^ and the location of the aneurysm in the present case can be recognized as either an AAA or a median sacral artery aneurysm. Either way, a stent graft was the first choice to treat the aneurysm in the present case, considering the patient’s general status.

In a literature review from 1999 to 2014, Orion et al. reported that the mortality rate was higher with endovascular therapy (19%) compared to open surgery (12%) when treating aorto-caval fistulas with AAAs.^5)^ Akwei et al. reported four cases of endovascular repair for aorto-caval fistula associated with AAA, and 75% of cases died because of failure in early diagnosis.^6)^ It has also been reported that early diagnosis improved the mortality after endovascular treatment to 3.8%.^5)^ It was also challenging to diagnose the present case as an AVF due to its uncommon presentation. The AVF was suspected on enhanced CT; however, a definitive diagnosis was made using 4DCT, confirming the fistula communicating between the AAA and the left iliac vein. Goto et al. reported that a multidetector row CT was useful in finding the fistula in AVF cases.^7)^ 4DCT also had the benefit of visualizing a sequential change from light to deep enhancement in the left iliac vein. In the present case, it was difficult to diagnose the location of the fistula because the aneurysm had an irregular margin, and 4DCT was more useful than three-dimensional CT. Additionally, 4DCT revealed that the arterial flow in the left iliac vein was more strongly enhanced in the distal part than in the proximal part of the AVF due to the stenotic change formed by the aneurysmal pressure (Supplemental data). The arterial flow in the direction of the inferior vena cava was decreased, which could be the reason why the patient did not present the symptoms of heart failure.

Open surgery has classically been used to repair AVFs with high mortality rates.^1)^ Recently, endovascular therapy has been used more frequently for ruptured arterial disease. A presence of an endoleak remains to be the main problem after inserting a stent graft. The endoleak rate after endovascular management of an AAA with aorto-caval fistula was estimated to be 15–50%, and the rate of fistula persistence was 9%.^8)^ Since the risk of endoleak in the present case was considered high, the aneurysmal sac was embolized with Impede-FX after stent graft insertion. Unfortunately, given the emergent situation, the median sacral artery was not identified preoperatively, and sac embolization was the first choice management to prevent an endoleak. Plugging the median sacral artery, if recognized preoperatively, is considered the better choice. No endoleak was observed in the final aortography.

The left iliac vein was covered using Viabahn VBX as off-label use in Japan. Zahradnik et al. reported that the Viabahn stent graft was valuable for repairing the left common iliac vein injured iatrogenically.^9)^ A few reports have argued the benefit of stent grafts for injured or perforated veins. However, the advantages of the Viabahn or Viabahn VBX for venous use should be further verified prior to allowing regular use in emergency situations. Cilostazol was reported to be effective in preventing stenosis of polytetrafluoroethylene-covered stents in veins.^10)^ Cilostazol was added to the present case, and the Viabahn VBX inserted in the left iliac vein was patent 1 month after surgery. This case should be followed up carefully, with emphasis on the CT findings.

## Conclusion

An AAA with AVF is a differential diagnosis of leg edema. 4DCT has the benefit of visualizing a sequential change in the vein, enabling diagnosis of the AVF. The use of stent graft for injured veins should be verified further.

## Consent

Informed consent was obtained from the patient for publication of the case report and the accompanying images. The Ethical Guidelines for Medical and Health Research Involving Human Subjects is applicable to this case report because of using the unapproved medical supply (intravenous use of the Viabahn VBX). Therefore, an ethical approval, which was issued by the ethical committee of Tokai University Hachioji Hospital, was obtained on July 29, 2022 (approval number 22-01).

## Supplementary Information

Supplementary movie is available at the online article sites on J-STAGE and PMC. Supplemental data: Movie of the four-dimensional reconstruction. First half: Arterial and venous flows are described in red and blue, respectively. The contrast medium flows from the aneurysm into the left iliac vein through the fistula. Enhancement of the left iliac vein and inferior vena cava immediately occurs after aortic enhancement. Second half: Arterial blood flow is described in red and which flows into the left iliac vein through the fistula. The arterial flow in the left iliac vein is recognized more strongly in the distal part than in the proximal part of the AVF.
